# Eighth ISABS Conference on Forensic, Anthropologic and Medical Genetics and Mayo Clinic Lectures on Translational Medicine, Split, Croatia, June 24-28, 2013

**DOI:** 10.3325/cmj.2013.54.217

**Published:** 2013-06

**Authors:** Dragan Primorac, Moses Schanfield, Stanimir Vuk-Pavlović

**Affiliations:** ISABS Conferences Co-Directors *draganprimorac2@gmail.com*

“… *and you shall see my back* …” (Ex. 33:23)

This issue of the *Croatian Medical Journal* is the ninth special issue over the past sixteen years containing articles prepared for the biennial conferences of the International Society for Applied Biological Sciences (Zagreb). Sustaining the conferences in the face of adverse economic situation in Croatia is an accomplishment that invites citing statistics: the number of speakers, topics, attendees, and their home countries, and so on. We have certainly been guilty of this in the past, but now it may be more appropriate to “see the back” and assess what ISABS, through the past conferences and other activities has accomplished for the advancement of applied genetics and experimental medicine in Croatia and beyond (1,2).

ISABS arose from the need for forensic evidence and identification of victims of the war of Yugoslav disintegration (1991-1995) and on the foundations laid by the expertise developed through the process (3-8). Early on, it became clear that modern forensic techniques of molecular biology are not dissimilar to those employed in modern medical diagnostics and anthropology. Consequently, from the very beginning ISABS has tried to stimulate the crosstalk of these disciplines by holding conferences that concomitantly covered all three. This crosspollination proved successful, as evidenced by the growing and increasingly international attendance of the conferences over the years.

The conferences have not been the goals unto themselves. A critical focus has been education; thus, the conferences have had a partial character of summer schools, both in maintaining a core of regular speakers and in attracting students and young investigators. Hence, each conference awarded the Young Investigator Awards to the best scientific presentations and invited the speakers to be available for unstructured and informal interactions with the attendees.

The results of the almost two-decade long effort, starting way before the first conference in 1997 and the formal establishment of ISABS in 2004, have been noticeable. The major formal result is the establishment in 2009 of the first graduate level program in the broader geographical region and the Center for Forensic Science at the University of Split. The two-year M.S. program covers the crime scene investigation, forensic toxicology and DNA analysis, national security and forensic accounting. The program is conducted with the active participation of faculty from the Penn State University (University Park, Pennsylvania), University of New Haven (New Haven, Connecticut), and since recently George Washington University (Washington, District of Columbia). Despite the youth of the program, its graduates have already made a major impact on the forensic practice in the region.

ISABS has played an important role in reaching consensus on important regulatory initiatives. For example, one such initiative pertains to the concordance of the pertinent Balkan databases (9). Another initiative resulted in affiliating the Slavic Speaking Working Group for Forensic Genetics (SSWFG) with ISABS (*http://www.isabs.hr/index.php/sswgfg-isabs/history-of-sswgfg-isabs*).

No less important have been numerous international collaborations in forensic science, molecular anthropology, and translational medicine stimulated by personal interactions forged at ISABS conferences. They have been effectuated through postdoctoral studies, short-term exchange, and data pooling. Another tangible testament of the catalytic role of ISABS is the compendium entitled “Forensic DNA Application: An Interdisciplinary Perspective” to be published by the CRC Press (Taylor and Francis Group) by the end of 2013. The book – conceived at the Sixth ISABS Conference and edited by D. Primorac and M. Schanfield – provides a thoroughly comprehensive and up-to-date review of forensic genetics through contributions by 51 authors from major centers of forensic research and practice worldwide.

Recently, ISABS mediated the formation of international consortia for joint applications to the Framework Program 7 of the European Union and other sources of funding; already such initiatives bore the first tangible results. Finally, as this very issue attests, ISABS-fostered interactions result in important scientific publications. We are excited by the momentum ISABS has generated and look forward to the future of the Society.
